# The synthetic peptide CIGB-300 modulates CK2-dependent signaling pathways affecting the survival and chemoresistance of non-small cell lung cancer cell lines

**DOI:** 10.1186/s12935-017-0413-y

**Published:** 2017-03-31

**Authors:** Stéfano M. Cirigliano, María I. Díaz Bessone, Damián E. Berardi, Carolina Flumian, Elisa D. Bal de Kier Joffé, Silvio E. Perea, Hernán G. Farina, Laura B. Todaro, Alejandro J. Urtreger

**Affiliations:** 1 Universidad de Buenos Aires, Instituto de Oncología “Ángel H. Roffo”, Área Investigaciones, Av. San Martín 5481, Buenos Aires, Argentina; 2 CONICET, Buenos Aires, Argentina; 3 Laboratorio de Oncología Molecular, División de Productos Farmacéuticos, Centro de Genética Ingeniería y Biotecnología (CIGB), Havana, Cuba; 4 Laboratorio de Oncología Molecular, Universidad Nacional de Quilmes, Bernal, Buenos Aires, Argentina

**Keywords:** CK2, CIGB-300, NSCLC, NF-κB

## Abstract

**Background:**

Lung cancer is the most frequently diagnosed cancer and the leading cause of cancer-related deaths worldwide. Up to 80% of cancer patients are classified as non-small-cell lung cancer (NSCLC) and cisplatin remains as the gold standard chemotherapy treatment, despite its limited efficacy due to both intrinsic and acquired resistance. The CK2 is a Ser/Thr kinase overexpressed in various types of cancer, including lung cancer. CIGB-300 is an antitumor peptide with a novel mechanism of action, since it binds to CK2 substrates thus preventing the enzyme activity. The aim of this work was to analyze the *effects* of CIGB-300 *treatment* targeting CK2-dependent signaling pathways in NSCLC *cell lines* and whether it may help improve current chemotherapy treatment.

**Methods:**

The human NSCLC cell lines NCI-H125 and NIH-A549 were used. Tumor spheroids were obtained through the hanging-drop method. A cisplatin resistant A549 cell line was obtained by chronic administration of cisplatin. Cell viability, apoptosis, immunoblotting, immunofluorescence and luciferase reporter assays were used to assess CIGB-300 effects. A luminescent assay was used to monitor proteasome activity.

**Results:**

We demonstrated that CIGB-300 induces an anti-proliferative response both in monolayer- and three-dimensional NSCLC models, presenting rapid and complete peptide uptake. This effect was accompanied by the inhibition of the CK2-dependent canonical NF-κB pathway, evidenced by reduced RelA/p65 nuclear levels and NF-κB protein targets modulation in both lung cancer cell lines, as well as conditionally reduced NF-κB transcriptional activity. In addition, NF-κB modulation was associated with enhanced proteasome activity, possibly through its α7/C8 subunit. Neither the peptide nor a classical CK2 inhibitor affected cytoplasmic β-CATENIN basal levels. Given that NF-κB activation has been linked to cisplatin-induced resistance, we explored whether CIGB-300 could bring additional therapeutic benefits to the standard cisplatin treatment. We established a resistant cell line that showed higher p65 nuclear levels after cisplatin treatment as compared with the parental cell line. Remarkably, the cisplatin-resistant cell line became more sensitive to CIGB-300 treatment.

**Conclusions:**

Our data provide new insights into CIGB-300 mechanism of action and suggest clinical potential on current NSCLC therapy.

## Background

Protein kinases are critical components of signaling pathways as they can promote or repress gene expression through reversible phosphorylation of target proteins [1]. Altered levels or activity of kinases may have dramatic effects on the control of cell growth, proliferation and apoptosis resulting in a high variety of human diseases including cancer [2].

The protein kinase CK2, formerly known as casein kinase 2, phosphorylates serine or threonine residues within acidic domains regulating a wide range of cellular processes. CK2 is probably the most pleiotropic protein kinase known, with more than 300 substrates already identified [3]. Related to cancer biology, CK2 phosphorylates many transcription factors, proto-oncoproteins and tumor suppressor proteins. In fact, the over-expression of CK2 catalytic subunits exacerbates the tumor phenotype, consistent with the rising concept that CK2 plays a general role as anti-apoptotic agent [4]. On the other side, blocking its expression is associated with the induction of apoptosis of both in vivo and in vitro [5]. Thus, CK2 could be considered a hallmark of tumor progression. CK2 is frequently overexpressed in various types of human cancer, including lung cancer [6], and its overexpression can cause mammary tumors [7] and lymphomas [8].

It has been recently proposed that CK2-mediated phosphorylation could function as a *druggable* target to treat cancer [9]. Different groups have tried to manipulate CK2 biochemical properties by targeting the ATP-binding site, the catalytic (α or α′) or regulatory subunits (β) of the holoenzyme (α α′/β β) or gene expression using antisense oligonucleotides [10, 11]. In this work we used the CIGB-300, a synthetic peptide developed following an innovative approach in order to target the phosphoaceptor site on the CK2 substrates rather than the enzyme per se, unlike most CK2 inhibitors [12].

Lung cancer is the most frequently diagnosed cancer and the leading cause of cancer-related deaths worldwide [13]. Up to 80–85% of lung cancers are classified as non-small-cell lung cancer (NSCLC). Surgical resection is the most potentially curative therapeutic modality for this disease. Cisplatin-based neoadjuvant (cisdiammine-dichloro-platinum) and/or adjuvant chemotherapy may provide an additional benefit to Stage II–IIIA patients and chemotherapy has produced short-term improvement in patients with advanced NSCLC [14, 15]. However innate and acquired resistance to cisplatin has become a major challenge in the management of lung cancer patients, indicating that it is imperative the development of new drugs with different mechanisms of action.

The lack of therapeutic alternatives, together with the knowledge that NSCLC overexpress CK2, make lung malignancies strong candidates for CIGB-300 treatment.

CIGB-300 is a proapoptotic peptide with established antiproliferative activity in vitro affecting transformed cells of different origin [16] including NSCLC. However, the subsequent events that lead tumor cells death remain far to be fully elucidated.

Studies in Drosophila have implicated CK2 in the Wnt pathway involved in embryonic development. In addition, Wnt pathway is increasingly recognized to play a role in cancer development, through modulation of genes encoding β-CATENIN itself or its regulators. In the absence of Wnt ligands, the β-CATENIN is phosphorylated at its N-terminus region by a protein complex, inducing its destruction by the proteasome [17]. CK2 is able to phosphorylate several proteins of this destruction complex favoring its disruption, consequently increasing the levels of free β-CATENIN in the cytoplasm. Furthermore, CK2 is able to phosphorylate β-CATENIN Thr393 increasing its stability [18]. Altogether these two processes favor the increased levels of β-CATENIN in the nucleus where it acts as a transcription factor, favoring the expression of several proteins involved in cell proliferation and apoptosis resistance [19].

NF-κB activation is a common event in cancer due to its antiapoptotic activity and pro-proliferative functions [20]. NF-κB is a dimeric transcription factor formed by p50, p52, p65/relA, relB, and c-rel subunits. Functional activation of NF-κB requires the separation from its inhibitor (IκB) in order to translocate to the nucleus and activate the transcription of target genes [21]. The loss of IκB occurs through a multistep process which includes phosphorylation signals, ubiquitination and finally proteasomal degradation. Several kinases can start this degradation cascade, including CK2 [22]. Classical NF-κB activators induce N-terminal phosphorylation, while CK2 induces an alternative C-terminal phosphorylation on Ser529, considered as a non-classic activation, also related to IκB degradation [23]. Moreover, CK2 also acts at multiple levels in NF-κB activation, as it targets not only IκB, but also other upstream IκB kinases [24] and p65 itself [25, 26].

As mentioned above, cisplatin response is not durable and lead to resistance, remaining as the main challenge in the treatment of NSCLC. Activation of NF-κB has been reported to be associated to cisplatin-induced cell resistance among other specific molecules [27]. In fact, the interaction between CK2 and chemoresistance has never been analyzed before.

A common denominator of the above mentioned signaling pathways is the proteolytic degradation mediated by the proteasome. 26S proteasome results from the association between the 19S regulatory particle to the 20S core which presents proteolytic properties. This proteasome conformation plays a major role in protein degradation by both ubiquitin-dependent and ubiquitin-independent mechanisms. CK2 is known to co-purify with 20S proteasome preparations [28] and it has been also described as responsible for its phosphorylation mapped in the residues Ser-243 and Ser-250 close to the C-terminus [29]. On NF-κB signaling pathway proteasome is responsible not only for IκB degradation, but also is essential for the attenuation of p65 nuclear levels [30].

Among the many roles displayed by CK2, modulation of above mentioned pathways could be critical for the control of tumor development and dissemination. Intrinsic CIGB-300 features make this synthetic peptide not only a powerful tool for dissecting the biological functions of CK2, but also an important anti-cancer drug. In this paper we demonstrate that CIGB-300 induces a significant anti-proliferative response both in two- and in three-dimensional lung cancer cellular models by affecting key signaling pathways associated with malignant progression. Moreover, this peptide showed improved effectiveness in a chemoresistance model associated with NF-κB inhibition. Altogether this new evidence indicates that CIGB-300 may become a new strategy for the treatment of lung cancer patients.

## Methods

### Peptide synthesis

CIGB-300 and CIGB-300-biotin-conjugated were synthesized as previously described by Perea et al. [12]. Briefly, the peptides were synthesized on solid phase and purified by reverse-phase high-performance liquid chromatography. The catalytic peptide sequence was fused to the TAT fragment of the HIV-1 virus, which confers the compound the ability to pass through the cell membrane [31, 32]. Purity was verified by mass spectrometry.

### Reagents and antibodies

Medium for cell culture was obtained from Life Technologies Inc. (Rockville, MD,USA). Fetal bovine serum (FBS) was from Internegocios (Buenos Aires, Argentina). Acrylamide and all other reagents for polyacrylamide gel electrophoresis were obtained from Bio-Rad (Richmond, CA, USA). Phorbol 12-myristate 13-acetate (PMA) and 4,5,6,7-Tetrabromo-2-azabenzimidazole (TBB) were purchased from Sigma-Aldrich Co (St. Louis, MO, USA). Recombinant human tumor necrosis factor-alpha (TNFα) was purchased from ImmunoTools (Friesoythe, Germany). Bortezomib (VELCADE^®^) was purchased from Millennium Pharmaceuticals (Cambridge, MA, USA) and cisplatin (MARTIAN^®^) was purchased from Kampel Laboratory (Buenos Aires, Argentina). Anti-NF-κB p65 (D14E12 XP^®^, 1:1000) was purchased from Cell Signaling (Danvers, MA, USA), β-CATENIN antibody (#610,153, 1:1.000) was from BD Transduction Laboratories (San Jose, CA, USA), Horseradish peroxidase-conjugated anti-mouse (1:5000), Mouse monoclonal anti c-MYC (sc-40, 1:100) and Mouse monoclonal anti CYCLIN E (sc-247, 1:200) were from Santa Cruz Biotechnology (Dallas, TX, USA). Anti-rabbit (1:5000) and β-ACTIN antibody (1:10.000) were from Sigma-Aldrich Co. Monoclonal anti PSMA3 (α7/C8) antibody (ab109532, 1:100), Rabbit Monoclonal anti NF-κB p65 (phospho S529) (ab109458, 1:1000) and Rabbit polyclonal anti BAX (ab69643, 1:1000) were from Abcam (Cambridge, MA, USA), Streptavidin-FITC (SNN1008, 1:300) and CYCLIN D1 antibody (RB-9041, 1:10000) were from Invitrogen-Thermo Fisher (Waltham, MA), Goat Anti-Rabbit IgG H&L Alexa Fluor^®^ 647 (ab150113, 1:250) and Goat Anti-Rabbit IgG H&L Alexa Fluor^®^ 488 (ab150079, 1:1000) from Abcam. Hybond-P membranes for blotting and chemiluminescence reagents (ECL) were from Amersham-GE Healthcare (Buckinghamshire, UK).

### Cell lines and culture conditions

We used two human non-small cell lung cancer (NSCLC) derived cell lines: the adenosquamous cell carcinoma NCI-H125 (CRL-5801) and the adenocarcinoma NIH-A549 (CCL-185). The murine L Wnt-3A cell line (CRL-2647) was used in order to produce conditioned media (CM) containing the wnt3a factor, following ATCC’s instructions. All cell lines were obtained from ATCC. H125 was grown in RPMI medium supplemented with 10% FBS and 80 µg/ml gentamycin. A549 and L cells were grown in DMEM media, with the above mentioned supplementation. All cell lines were cultured at 37 °C in a humidified air atmosphere with 5% CO_2_.

### Treatments

For Wnt/β-CATENIN pathway studies, H125 sub-confluent monolayers were first incubated during 24 h with 50% of CM containing the wnt3a factor and then treated for another 6 h with a low-lethal dose of CIGB-300 (IC_25_) or during 2 h with the CK2 competitive inhibitor TBB (25 μM) as a control. For NF-κB studies, the H125, A549 or A549-cispR cells were incubated for 15 or 45 min respectively with a low-lethal dose CIGB-300 (IC_25_) with or without PMA (16 nM), TNFα (10 ng/ml) or the IC_50_ of cisplatin (1.0 µM for H125 and 5.0 µM for A549). For the analysis of NF-κB downstream targets, H125, A549 or A549-Rcisp cells were incubated for 2 h with CIGB-300 (IC_50_). In the case of Bortezomib, cells were previously pretreated with 8 nM of this drug during 4 h.

### Cell viability and apoptosis determination

To assess the effect of CIGB-300 or cisplatin on cell viability, 1 × 10^4^ cells/well were seeded into 96 well plates in standard culture conditions. Twenty-four hours later cells were treated with different doses of CIGB-300 (25–400 μM) or cisplatin (0.3–30 μM) during 72 h. Cell proliferation was evaluated after 72 h using the MTS assay (Celltiter 96TM Non Radioactive Proliferation Assay, Promega), as described by the manufacturer. Data were analyzed using the R package “ic50” [33] in order to determine the IC_50_ values for each drug. Alternatively, proliferative potential was determined by assessing cell number. Briefly, cells were seeded onto 35 mm Petri dishes, treated and after 72 h wells were washed twice with PBS, trypsinized, centrifuged, resuspended in a proper volume and finally counted using an hemocytometer and trypan blue exclusion. For apoptosis determination, A549 and A549-cispR cells growing on glass coverslips were treated with 300 μM of CIGB-300 for 18 h and then stained with Acridine orange (10 μg/ml) and Ethidium bromide (10 μg/ml). Visualization was performed with a Nikon, Eclipse E400 epifluorescence microscope. Uniform green nuclei with organized structure were considered as live cells. Bright green or orange to red nuclei were classified as early or late apoptotic cells respectively. Uniformly orange to red nuclei with organized structure were ascribed as necrotic cells. The percentage of dead cells was determined for each cell line.

### 3D cultures and peptide internalization assay

Multicellular tumor spheroids were obtained through the hanging-drop method [34]. Growth kinetic was evaluated using spheroids formed from 1000 initial cells. For the 3D growth inhibition assay, each “drop” containing a single spheroid was placed in 500 μl of culture media supplemented with FBS onto 48 well plates on day 3. Two days later spheroids were treated or not with different doses of CIGB-300 (IC_25_ and IC_50_ obtained for monolayer cultures). Complete culture media, supplemented or not with CIGB-300 was replaced every 48 h. At the same time, images were taken using an optical light microscope. The area, circularity and roundness of spheroids were measured using a custom macro for ImageJ, and finally the volume was calculated. For peptide internalization kinetics, 2-day-old spheroids were treated with the biotinylated variant of CIGB-300 for 5–60 min. Spheroids were fixed in formaldehyde (4% in PBS) for 30 min and embedded in paraffin. Sections of 5 μM were obtained using microtome on polylysine-coated slides. Finally, immunocytochemistry was performed using a Streptavidin–biotin method with the VECTASTAIN Elite ABC KIT from Vector Laboratories (Burlingame, CA, USA) following manufacturer’s instructions. Sections were counter-stained with Mayer’s hematoxylin. Images were taken using a light microscope, at 400× magnification. Immunoreactive staining was quantified transforming each image into polar coordinates and using the color detection tool of the ImageJ plugin Immunohistochemistry Image Analysis Toolbox [35].

### Separation of nuclear and cytoplasmic fractions

In order to determine β-CATENIN expression levels, cytoplasmic proteins were extracted using saponin buffer. Briefly, monolayer cultures were incubated for 20 min with ice-cold 0.1% saponin lysis buffer (25 mM Hepes, 75 mM potassium acetate and 0.1% saponin plus phosphatase and protease inhibitors). The extraction procedure was carried out twice and the extracts were pooled prior to their centrifugation. For NF-κB detection, nuclear and cytoplasmic proteins were separated by differential centrifugation following Abcam protocol. Briefly, semiconfluent monolayers were washed twice with ice-cold PBS and then collected in 250 μl of Fractionation Buffer (HEPES 20 mM, KCl 10 mM, MgCl_2_ 2 mM, EDTA 1 mM and 2-Mercaptoethanol 1 M) with protease and phosphatase inhibitors by scrapping with a Teflon scrapper. Cell suspensions were passed 10 times through a 25 gauge needle, and leaved on ice for 20 min. Then samples were centrifuged at 700*g* for 5 min. The pellets, which contain nuclei, were washed repeating all the procedure, and finally resuspended in Nuclear Buffer (Fractionation Buffer plus 10% glycerol and 0.1% SDS). Supernatants were centrifuged again at 10,000*g* for 5 min and the supernatant, containing the cytoplasm and membrane, was collected.

### Western blot analysis

Semiconfluent monolayers were washed twice with ice-cold PBS and then lysed with RIPA buffer (150 mM NaCl, 1% NP-40, 50 mM Tris–HCl pH 8.0, 1 mM EDTA, 0.5% deoxycholate) with protease and phosphatase inhibitors by scrapping with a Teflon scrapper. Samples were run in 10% SDS-PAGE, and the gels blotted to Hybond-P membranes. Membranes were blocked for 1 h in 5% skim milk in TBS, 0.1% Tween-20. Membranes were then incubated with the first antibody overnight at 4 °C, and with a secondary antibody coupled to horseradish peroxidase [1 h at room temperature (RT)]. Detection was performed by chemiluminescence. Bands were digitalized with a Photo/Analyst Express System (Fotodyne Inc. Hartland, WI, USA) and signal intensity was quantified with ImageJ software.

### NF-κB-dependent reporter gene expression

H125 cells were transiently co-transfected with NF-κB-RE-luc Luciferase Reporter Vector pGL4.32 and the Renilla Luciferase Control Reporter Vector pRL-TK (Promega, Madison, WI) in a 10:1 ratio, using Fugene (Promega) following manufacturer’s instructions. Briefly, 1 × 10^5^ cells/well were seeded onto 24-well plates and transfected the next day. Six hours after adding transfection reagents, cells were washed and complete media was added. After an overnight recovery, cells were treated for 6 h with PMA (16 nM), in presence or not of CIGB-300 (IC_25_) during the first hour. Finally cells were lysed and the luciferase activities were determined using the Dual-Luciferase Reporter Assay System (Promega) and normalized to the constitutive Renilla luciferase activity.

### Immunofluorescence microscopy

H125 cells growing on glass coverslips were treated with CIGB-300 for 15 min in presence or not of PMA, washed twice with PBS, fixed in ice cold paraformaldehyde, permeabilized and blocked with PBS containing 5% bovine serum albumin for 1 h at RT. p65 protein was detected by incubation with a specific primary antibody diluted in PBS containing 5% BSA, followed by an incubation with a secondary antibody coupled to Alexa 488 for 1 h at RT. Nuclei were counterstained with DAPI. Images were obtained using an epifluorescence microscope (Nikon, Eclipse TE2000), processed and analyzed using the ImageJ software. Nuclear intensity was measured taking into account the overlapping with DAPI staining. The number of nuclei with highly positive signal was counted and expressed as a percentage of the total intensity for each condition.

### Generation of a cisplatin resistant cell line

Cisplatin resistant A549 lung cancer cell line (A549-cispR) was obtained by the chronic administration of cisplatin IC_50_ (5.0 μM) during 6 months as previously described [36]. Parental A549 cells were maintained in culture during the same period.

### Drugs interaction analysis

To determine the combination index (CI) of CIGB-300 and cisplatin, A549 and A549-cispR cells were treated with both drugs following a constant-ratio analysis. Briefly, drug mixture was serially diluted and added for 72 h to cells seeded onto a 96 well plate. Drugs and media were refreshed every 24 h. The combination ratio used was approximately equal to the IC_50_ ratio for each drug. Three points below and above the IC_50_ value were used for each mixture. Effects of all the mixture points were displayed using the form CI vs. Fa, where Fa is fraction affected and represents the respective proliferation inhibition parameters (e.g., a Fa of 0.5 is a proliferation inhibition of 50%). CI plot values were obtained from three different experiments using the Compusyn software [37].

### Proteasome activity determination

Proteasome activity was assessed in H125 cells using the Proteasome-Glo Chymotrypsin-Like Cell-Based Assay (Promega) following manufacturer’s instructions. Briefly, H125 cells were plated onto 96-well plates and 24 h later treated with CIGB-300 (30 min) or with Bortezomib (4 h). Briefly: 100 µl of Proteasome-Glo™ Cell-Based Reagent were added to each 100 µl of sample and incubated at RT for 10 min. Luminescence emission was measured with a Multimode Microplate Reader, Synergy luminometer (Biotek).

### Confocal immunofluorescence

H125 cells growing on glass coverslips were treated with biotin-conjugated CIGB-300 for 30 min, washed twice with PBS, fixed in ice cold paraformaldehyde, permeabilized and blocked with PBS containing 5% bovine serum albumin for 1 h at RT. α7/C8 protein was detected by incubation with a specific primary antibody diluted in PBS containing 5% BSA, followed by an incubation with a secondary antibody coupled to Alexa 647 and with Streptavidin-FITC conjugated for 1 h at RT. Nuclei were stained with DAPI. Images were obtained in a LSM 510 Meta confocal Zeiss microscope. Confocal images were processed for presentation with ImageJ. Background of each channel was subtracted. Pearson’s and Mander’s coefficients were calculated using the JACoP plugin [38]. Single cell analysis for 15–20 representative cells was performed on three independent experiments.

### Statistical analysis

Data obtained were evaluated for their statistical significance with the two-tail paired Student’s t test or analysis of variance (ANOVA) with post hoc corrections. Values were considered statistically significant at p below 0.05.

## Results

### CIGB-300 is able to penetrate into lung cancer three-dimensional spheroid cultures inhibiting their growth at the same effective dose than in monolayer cultures

**Fig. 1:**
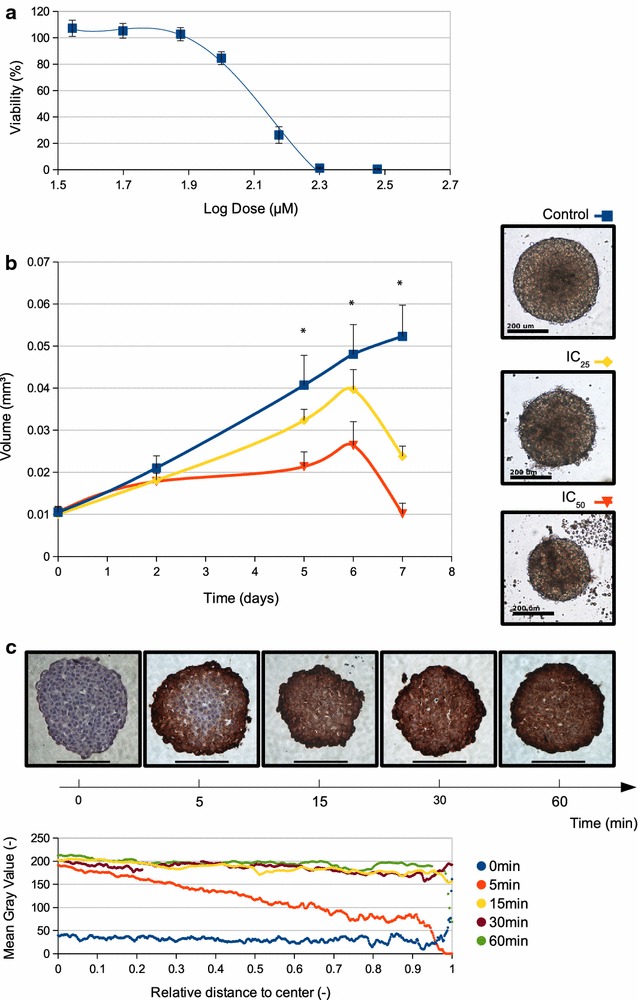
Anti-proliferative effect of CIGB-300 on 2D and 3D cultures. **a** IC_50_ determination for H125 monolayer cultures. Each value represents the mean ± SD of three independent experiments. **b** The IC_50_ dose obtained in H125 cells growing in monolayer was tested on multicelullar tumor spheroids. Each value represents the mean ± SD of three independent experiments. *p < 0.001 vs. H125 spheroids without treatment, 2-way ANOVA test (*left side*). Representative images for three different experiments were taken after 5 days of treatment, *scale bar* 100 μM (*right side*). **c** Internalization studies on H125 spheroids were performed treating cells from 5 to 60 min with a biotin-labeled CIGB-300 and visualized by immunocytochemistry. Representative images of three different experiments. Zoom 400×, *scale bar* 100 μm (*top*). Immunoreactive staining was quantified as a function of the distance to the spheroid center for each condition (*bottom*)

Previous reports describe the antiproliferative and pro-apoptotic effects of CIGB-300 on several cancer cell lines [39, 40]. In order to confirm the effect of CIGB-300 on NSCLC, H125 and A549 monolayers were treated with different doses of the peptide. Both cell lines responded to CIGB-300 with IC_50_ values of 124.2 μM and 271.0 μM (H125 and A549, respectively), H125 being the most susceptible and our first model choice for further mechanistic studies (Fig. 1a). Given multicellular tumor spheroids are a good in vitro model for drug delivery to early-stage avascular tumors as well as micrometastases, CIGB-300 effectiveness was also evaluated on three dimensional cultures. We tested CIGB-300 on H125 spheroids using the IC_25_ and IC_50_ doses previously determined for monolayers. After 5 days of treatment, the volume of CIGB-300-treated spheroids was significantly lower for both assessed doses compared with untreated controls. Moreover, on day 7, the volume of treated spheroids dropped markedly, even reaching initial volume values for IC_50_ treated spheroids (Fig. 1b). Thus, the peptide proved to be effective for both two- and three- dimensional cultures at equivalent doses. We hypothesized this could be related with CIGB-300 ability to penetrate into the spheroid compact structure, where most of the chemotherapeutic agent fail to accomplish a fast and effective entrance [41–44]. Therefore, H125 spheroids were treated with a biotinylated version of the peptide and tracked over time. Interestingly, a moving front was detected as soon as 5 min after initiation of the treatment and complete penetration into the spheroid core was observed at 60 min (Fig. 1c). Based on these results, a low-lethal (IC_25_: 60 μM for H125 and 150 μM for A549) and a high-lethal (IC_50_: 120 μM for H125 and 300 μM for A549) CIGB-300 doses were selected for use in the following experiments.

### CK2-dependent signaling pathways are affected by CIGB-300 treatment

Since CK2 has more than 300 intracellular substrates [45], several signaling pathways might be potentially altered by CIGB-300 [1]. In the present work we studied two of the more relevant due to their role on survival and apoptosis inhibition [46]: the canonical NF-κB and Wnt/β-CATENIN pathways.

**Fig. 2:**
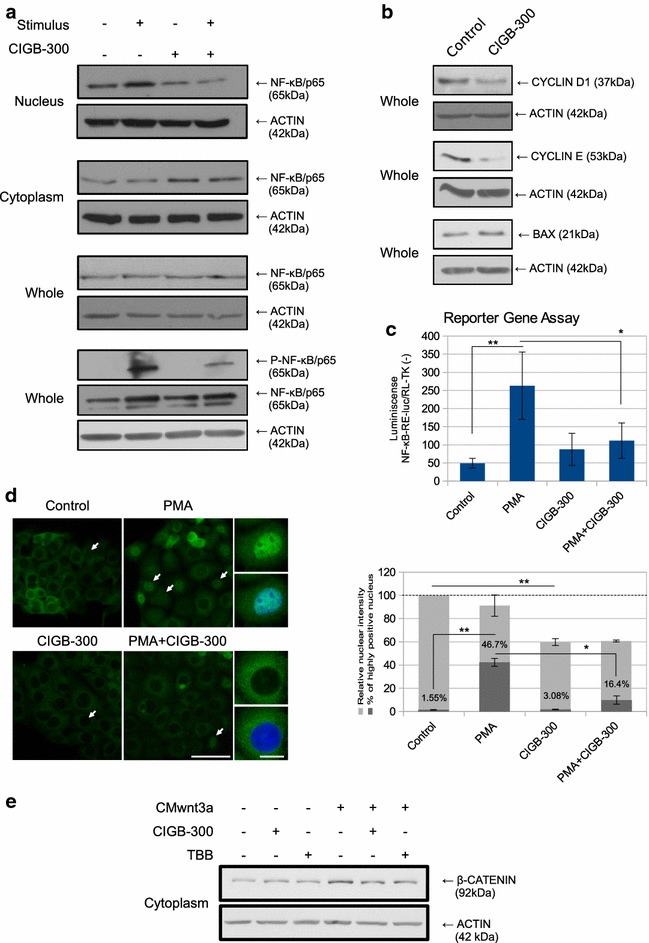
Effect of CIGB-300 on CK2-dependent signaling pathways. **a** Nuclear, cytoplasmic or whole cell extracts prepared from H125 cells treated with low-lethal doses of CIGB-300 in combination or not with an activating stimulus, were resolved on 10% SDS-PAGE and blotted with p65/RelA and P-p65 (Ser529) antibodies (50 μg protein/lane). ACTIN expression level was used as protein loading control. For the determination of p65 levels PMA was used as the activating stimulus, while TNFα was used for P-p65 determinations. Results are representative of three independent experiments. **b** Whole cell extracts prepared from H125 cells treated with CIGB-300, were resolved on 10% SDS-PAGE and blotted with CYCLIN D1, CYCLIN E and BAX antibodies (50 μg protein/lane). ACTIN expression level was used as protein loading control. **c** H125 cells were transiently co-transfected with pNF-κB-RE-luc and pRL-TK-luc vectors, treated with low-lethal doses of CIGB-300 in combination or not with PMA and luciferase activity was determined. Data were normalized to the constitutive Renilla luciferase activity and expressed as the mean ± SD. **p < 0.01 vs. PMA treated cells and *p < 0.05 PMA + CIGB-300 treated cells vs. PMA treated cells (one-way ANOVA test). Results are representative of three independent experiments. **d** H125 cells were treated with low-lethal doses of CIGB-300 in combination or not with PMA. Cellular distribution of p65 was visualized by immunofluorescence microscopy. Figure shows representative images of three independent experiments, *scale bar* 50 μm. *White arrows* indicate highly positive nucleus. Representative *insets* of cells treated with PMA in combination or not with CIGB-300 are shown, *scale bar* 5 μm (*left side*). Relative p65 nuclear intensity was measured and represented as a percentage of control cells (*light gray bars*). **p < 0.01 vs. CIGB-300 treated cells (one-way ANOVA test). The percentage of those cells with highly positive nucleus (*white arrows*) was also measured (*dark gray bars*). **p < 0.01 vs. PMA treated cells and *p < 0.05 PMA + CIGB-300 treated cells vs. PMA treated cells (one-way ANOVA test) (*right side*). **e** H125 cells were incubated with CM containing the wnt3a factor and then treated with low-lethal doses of CIGB-300 or TBB. Cytoplasmic protein content was separated by SDS-PAGE 10% and the membrane was blotted with anti β-CATENIN antibody (50 μg protein/lane). ACTIN expression level was used as protein loading control. Results are representative of three independent experiments

The canonical NF-κB pathway activation requires phosphorylation by CK2 to allow the nuclear translocation of the NF-κB complex. As shown in Fig. 2a, low-lethal dose of CIGB-300 reduced the nuclear levels of the p65/relA NF-κB complex subunit in H125 cells, while cytoplasmic p65 levels accumulated. This inhibitory effect was sustained even after the addition of PMA, an NF-κB activator. Consistently, the phosphorylation of the CK2 specific site in p65 induced by TNFα was reduced in the presence of CIGB-300 (Fig. 2a bottom, lane 2 vs. lane 4). In order to determine the extent of NF-κB inhibition after CIGB-300 treatment, the expression of several NF-κB downstream targets, such as CYCLIN D1, CYCLIN E and BAX, was evaluated by Western blot. As shown in Fig. 2b, both cyclins were downregulated whereas the proapototic protein BAX was upregulated. Moreover, we observed decreased NF-κB transcriptional response upon CIGB-300 treatment in the presence of PMA using a reporter gene assay (Fig. 2c). Finally, in line with the above described results, we could visualized by immunofluorescence the reduced p65 nuclear intensity after CIGB-300 treatment (Fig. 2d). PMA treated cells presented highly positive nuclei that were reduced upon combination with CIGB-300 (Fig. 2d, white arrows and representative insets). Taken together, all these evidences confirm the inhibition of NF-κB pathway by CIGB-300.

In the Wnt/β-CATENIN pathway, CK2 phosphorylates and stabilizes β-CATENIN allowing translocation to the nucleus and transcription of target genes [18]. Basal levels of cytoplasmic β-CATENIN were not reduced after CIGB-300 treatment. However, when the Wnt/β-CATENIN pathway was active, in response to the treatment with a CM containing soluble wnt3a factor, CIGB-300 partially reverted β-CATENIN cytoplasmic accumulation, suggesting the involvement of the peptide on the blockade of β-CATENIN signaling (Fig. 2b). Additionally, the competitive CK2 inhibitor TBB [47] produced similar effects on β-CATENIN accumulation than CIGB-300.

### Cisplatin-resistance is associated with NF-κB signaling on NSCLC cells

**Fig. 3:**
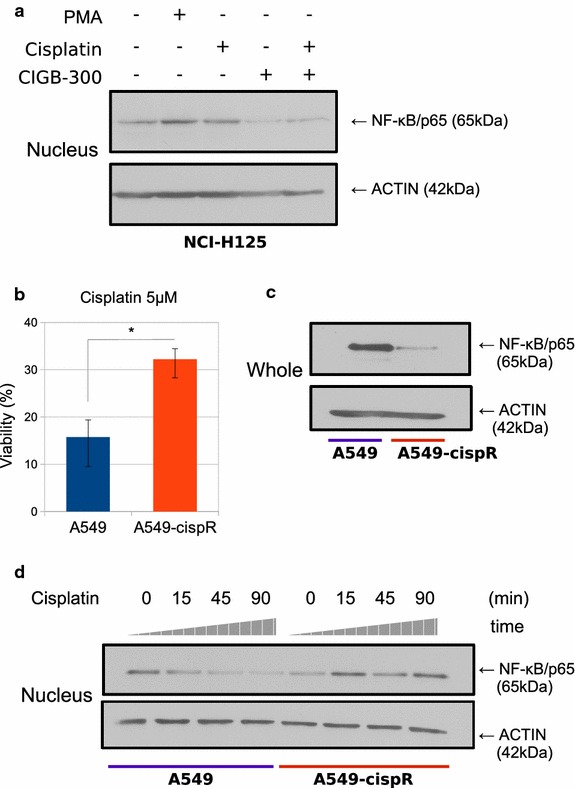
NF-κB signaling pathway status after cisplatin treatment. **a** H125 cells were treated with low-lethal doses CIGB-300 for 45 min in the presence or not of cisplatin. Nuclear extracts were prepared, resolved in 10% SDS-PAGE and blotted with anti p65/RelA antibodies (50 μg protein/lane). ACTIN expression level was used as protein loading control. Results are representative of three independent experiments. **b** A549 and A549-cispR cultures were treated with cisplatin and cell number was assessed 72 h later. Viability values were compared to those obtained in both cell lines without treatment, which were set as 100%. Data expressed as the mean ± SE are representative of three independent experiments. *p < 0.05 vs. the parental cell line (Student’s t test). **c** Whole cell lysates prepared from A549and A549-cispR cells were resolved on 10% SDS-PAGE and blotted with anti p65/RelA antibodies (50 μg protein/lane). ACTIN expression level was used as protein loading control. Results are representative of three independent experiments. **d** Nuclear extracts prepared from A549and A549-cispR cells treated with cisplatin were resolved on 10% SDS-PAGE and blotted with anti P65/RelA antibodies (50 μg protein/lane). ACTIN expression level was used as protein loading control. Results are representative of three independent experiments

It is known that cancer patients’ refractoriness to cisplatin-based chemotherapy may be associated with NF-κB signaling activation [48–50]. Since CIGB-300 prevented NF-κB nuclear translocation even in the presence of an activating stimulus, we explored in vitro whether its combination with cisplatin could generate some therapeutic benefit. In fact, H125 cells treated with cisplatin showed an important increase in nuclear p65 levels, which were comparable or even stronger than those displayed by the same cell line under PMA stimulus (Fig. 3a). Moreover, CIGB-300 was able to impair the increased p65 levels observed under the chemotherapeutic treatment (Fig. 3a, lane 5). These results prompted us to evaluate CIGB-300 effectiveness in a chemoresistant setting. To this end, we developed a cisplatin resistant variant of the NSCLC cell line A549. Chronic supplementation with the IC_50_ dose for cisplatin during 6 months allowed us to obtain the A549-cispR variant. As shown in Fig. 3b, after cisplatin treatment, the viability of the new resistant cell line was twofold higher than that in the parental cells, and these differences were sustained over time and even after two cycles of thawing/freezing, confirming cisplatin-acquired resistance. Surprisingly, A549-cispR expressed lower total p65 levels than A549 (Fig. 3c). Nevertheless, when both cell lines were treated with cisplatin they showed different NF-κB activation patterns. While parental cells decreased p65 nuclear levels after treatment, the cisplatin-resistant cell line showed higher levels (Fig. 3d).

### Cisplatin-acquired-resistance sensitizes to CIGB-300 treatment

**Fig. 4:**
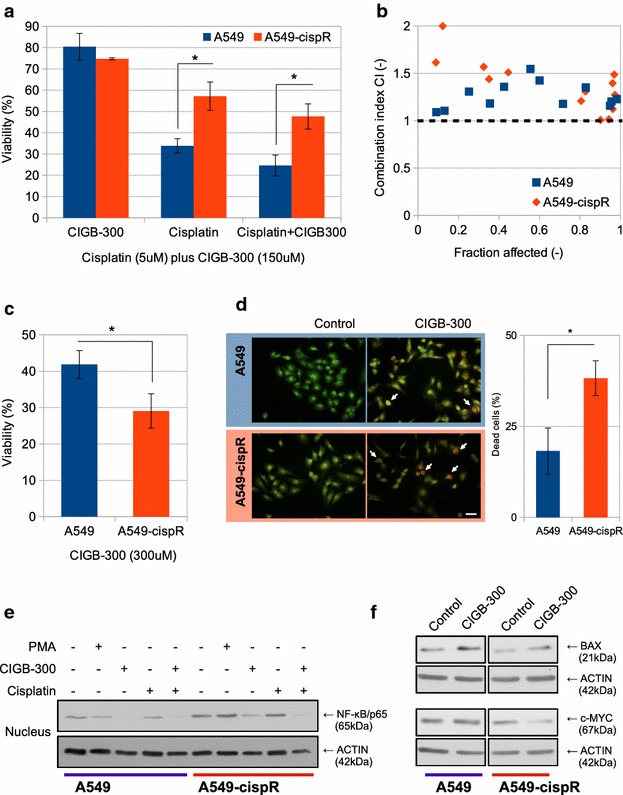
Evaluation of Cisplatin and CIGB-300 combined treatment in a chemo-resistant setting. **a** A549 and A549-cispR cells were treated with low-lethal doses of CIGB-300 (150 μM) and/or Cisplatin and cell number was assessed 72 h later. Viability values were compared to those obtained in both cell lines without treatment, which were set as 100%. Data represent the mean ± SE of three independent experiments. *p < 0.05 vs. the parental cell line (one-way ANOVA test). **b** Chou and Talalay combination index (CI) plot for CIGB-300 and Cisplatin values of three independent experiments. **c** A549 and A549-cispR cells were treated with a high dose of CIGB-300 (300 μM) during 72 h and cell viability was evaluated using the MTS assay. Viability values were compared to those obtained in both cell lines without treatment, which were set as 100%. Data represent the mean ± SE of three independent experiments. *p < 0.01 vs. the parental cell line (Student’s *t* test). **d** A549 and A549-cispR cells were treated with a high dose of CIGB-300 (300 μM) during 18 h and apoptosis induction was visualized by an acridine orange/ethidium bromide double staining assay. Figure shows representative images of three independent experiments (*left side*). The number of dead cells was quantified and expressed as a percentage of the total cell number for each cell line (*right side*). Data represent the mean ± SE of three independent experiments. *p < 0.05 vs. the parental cell line (Student’s t test). **e** A549 and A549-cispR cells were treated with 150 μM of CIGB-300 for 90 min in the presence or not of cisplatin. PMA was used as control. Nuclear extracts were prepared, resolved in 10% SDS-PAGE and blotted with anti p65/RelA antibodies (50 μg protein/lane). ACTIN expression level was used as protein loading control. Results are representative of three independent experiments. **f** Whole cell extracts prepared from A549 and A549-cispR cells treated with 300 μM of CIGB-300, were resolved on 10% SDS-PAGE and blotted with BAX and c-MYC antibodies (50 μg protein/lane). ACTIN expression level was used as protein loading control

Based on the previous evidence that cisplatin resistance acquisition was accompanied with NF-κB activation, and given that CIGB-300 blocked the cisplatin response, we evaluated the effect of the combination treatment on both resistant and parental A549 cell lines. When we combined low-lethal CIGB-300 dose with cisplatin we could observe a higher but non-significant response than with the single treatments (Fig. 4a). To determine the kind of interaction between these drugs, we calculated the combination index (CI) using Chou and Talalay method [37]. Neither parental nor resistant cell lines present an agonist or synergistic interaction along all the fraction affected values analyzed (CI > 1) (Fig. 4b). Nevertheless, a higher response to CIGB-300 was observed in the cisplatin resistant A549 cell line when we used higher CIGB-300 doses (Fig. 4c). The sensitization to CIGB-300 was also corroborated by a significant IC_50_ reduction (A549: 271 ± 16 μM vs. A549-cispR: 249 ± 15 μM; *T* test p < 0.01) and apoptosis assessment by acridine orange/ethidium bromide double staining, where the number of dead cells on A549-cispR was significantly increased, presenting in both cell lines early and late apoptosis, as well as necrosis in a minor proportion (Fig. 4d). In order to explore the molecular mechanisms involved in the response of both cell lines to CIGB-300, we analyzed the modulation of nuclear p65 levels as well as NF-κB downstream targets. In both parental and resistant A549 variants, the treatment with CIGB-300 reduced the p65 nuclear levels, even in presence of cisplatin (Fig. 4e). Regarding downstream targets, the proapoptotic protein BAX levels increased in both cell lines after CIGB-300 treatment. Interestingly, c-MYC protein, which is involved in proliferation, was distinctively inhibited only on the A549-cispR cell line (Fig. 4f).

### NF-κB is modulated through enhanced proteasome activity

**Fig. 5:**
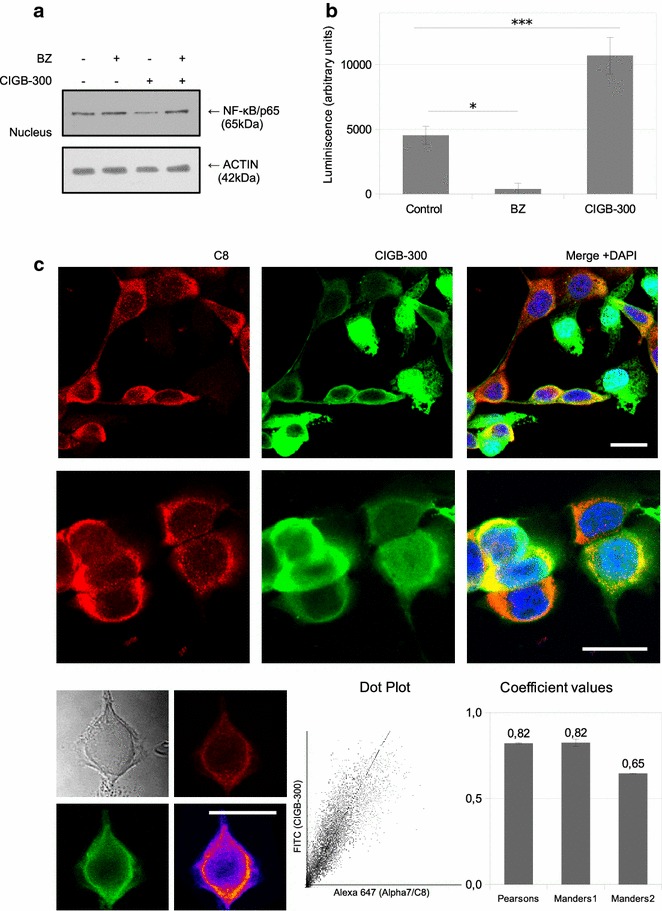
Association between NF-κB modulation and proteasome activity. **a** H125 cells pretreated with a non-cytotoxic dose of Bortezomib were exposed to CIGB-300. Nuclear extracts were prepared, resolved in 10% SDS-PAGE and blotted with anti p65/RelA antibodies (50 μg protein/lane). ACTIN expression level was used as protein loading control. Results are representative of three independent experiments. **b** Proteasome activity determination following the Proteasome-Glo™ cell-based assay for H125 treated with CIGB-300 or Bortezomib (Bz) as positive control. Data was expressed as the mean ± SE of three independent experiments. *p < 0.05 vs. Bz treated cells and ***p < 0.001 vs. control treated cells (one-way ANOVA test). **c** Cellular distribution of CIGB-300 (*green*, FITC) and α7/C8 protein (*red*, Alexa647) was visualized by confocal microscopy (*top*). Figure shows representative images of three independent experiments. Single cell analysis of Pearson and Manders coefficients were performed, as well as a representative colocalization map and a dot plot for both probes (*bottom*). Coefficient values are expressed as the mean ± SE of three independent experiments

To investigate how CIGB-300 inhibits NF-κB, we studied some of the main molecular mechanisms involved in the downregulation of p65 nuclear levels. Neither the IκB inhibitor nor the basal phosphorylation on CK2-specific p65 site were affected after CIGB-300 treatment, indicating that other processes are involved (data not shown). We could determine that the inhibition of p65 nuclear translocation, induced by CIGB-300, was prevented by a pretreatment with the proteasome inhibitor Bortezomib (Fig. 5a, lane 4), thus indicating that proteasome activity is necessary for the effect observed on NF-κB modulation. Indeed, we found that CIGB-300 increased proteasomal activity in H125 cells under the same conditions than inhibit nuclear p65 levels (Fig. 5b). Then we investigated which proteasomal proteins could be involved. We focused in α7/C8, a member of the proteasome alpha-ring, since previous evidence showed that this CK2 substrate co-precipitated with CIGB-300 in a pull-down assay (Perea and collaborators, unpublished data). Hence, we explored CIGB-300 and α7/C8 cell distribution by immunofluorescence. The protein α7/C8 was localized only in cytoplasm. In contrast, CIGB-300 was found both in the nuclear and cytoplasmic compartments. While one-third of the cells presented strong mark of CIGB-300 in the nucleus, the rest showed cytoplasmic signal, mainly located in the perinuclear area. Remarkably, in the latter group of cells, high CIGB-300 co-occurrence with α7/C8 was evidenced. More than 80% of α7/C8 is coincident with CIGB-300 presence (Fig. 5c), confirming the association between CIGB-300 and the CK2 proteasomal substrate α7/C8.

## Discussion

In this paper, we demonstrate that CIGB-300 induces a significant anti-proliferative effect on 2D and 3D NSCLC cultures. Both cell lines studied exhibited a response to CIGB-300. The differences in the IC_50_ values between them may be linked to p53 status: while A549 is a p53 wild-type cell line, H125 cells express a non-functional p53 variant. Drug-treated spheroids presented a lower growth rate from day 1 to 6 and suffered a great shrinkage at day 7, which may be caused by the induction of cell death as a consequence of 5 days of treatment. Remarkably, CIGB-300 treatment on spheroids cultures resulted effective using the same doses for monolayer cultures treatments. Most cancer drugs show less growth inhibition when cells are grown as multicellular aggregates [51, 52]. Several factors are responsible for this difference, including drug penetration capacity and survival-signaling activation, prompted by cell–cell interactions such as PI3 K/Akt, NF-κB and Stat3 [53]. Doublier and col. have demonstrated that mammary tumor spheroids were less sensitive to doxorubicin and presented reduced drug uptake than monolayer cultures [54]. Furthermore, Fayad and col. found that cisplatin only affect the peripheral 30 μm cell layer of a three-dimensional carcinoma model, even in presence of high doses and after 96 h of treatment [55]. In this context, CIGB-300 proved to be effective both in 2D and 3D cultures and this is consistent with the fact that internalization in 3D structures was rapid and complete, probably due to its peptidic nature and the presence of the cell-penetrating peptide TAT [31].

To our knowledge, this is the first work studying CIGB-300 effects on *pathways linked to CK2 signaling on lung cancer*, thus extending previous findings on CIGB-300 mechanism of action [40]. In this regard, the canonical NF-κB pathway appeared as the most relevant, where nuclear RelA/p65 levels were severely and significantly reduced by CIGB-300 in all the analyzed lung cancer cell lines. Supporting this finding, the NF-κB downstream target and proaptotic protein BAX was increased after CIGB-300 treatment in the same cell lines, while the expression of other target proteins such as cyclins (D1 and E) and c-MYC appeared downregulated on H125 and A549-cispR respectively. In addition, transcription of the NF-κB Response Element was conditionally affected by CIGB-300, as well as the number of positive p65 nuclei visualized by immunofluorescence microscopy. However, we did not observe differences after CIGB-300 treatment on the transcription levels of the NF-κB-RE-luc in the absence of PMA, indicating that a context of NF-κB stimulation might be necessary to detect the modulation on the transcription of the luciferase gene.

In line with our findings, recent reports have shown that the CK2 inhibitor CX-4945 impairs NF-κB promoter activity in human head and neck squamous cell carcinoma lines [56] and reduces p65 phosphorylation on Ser529 (a CK2 target site) in multiple myeloma cells isolated from patients [57]. These studies also highlight that p65 is one of the main targets of CK2 inhibitors. We also found that CIGB-300 altered the phosphorylation of p65 on Ser529 caused by TNFα. This evidence reinforce our results and also suggest a direct implication of CIGB-300 on the NF-κB signaling, but not explain the reduced p65 nuclear levels caused by the treatment in the absence of TNFα, where proteasome-mediated degradation seems to be involved.

In addition, the relevance of NF-κB activation on lung cancer metastasis has been well documented [58, 59]. On this regard, our findings are in agreement with those presented by Benavent and col. who recently showed that CIGB-300 was capable of reducing tumor cell dissemination and colonization into the lung, thus displaying a powerful antimetastatic effect [60].

In contrast, neither the peptide nor the classical CK2 inhibitor TBB affected the cytoplasmic basal levels of β-CATENIN, indicating that CIGB-300 anti-proliferative effect does not depend on the Wnt/β-CATENIN pathway under these conditions. Nevertheless, this may be due to the low basal β-CATENIN levels in these cells, which could be modulated only upon pathway activation with a Wnt ligand such as wnt3a factor. Therefore the CIGB-300 effect on Wnt/β-CATENIN pathway might be relevant in tumor cells with high constitutive levels of β-CATENIN, due to constitutive or transient microenvironmental activation [61].

Proteasome degradation complex has become relevant in the last years as a promising *druggable* target following the clinical success of Bortezomib [62]. Here we propose a new CIGB-300 target, the CK2 substrate and member of the proteasome proteolytic core 20S: the subunit PSMA3 (originally named as α7/C8). This is based in our findings of CIGB-300 co-occurrence with α7/C8 together with perturbed proteasome activity. Moreover, modulation of the proteasome machinery with analogous peptides has been described by Zanin and col [63]. The fact that proteasome activation is necessary for nuclear p65 inhibition suggests an interesting link between both CIGB-300 effects and deserves further analysis.

It has been previously described that CIGB-300 co-localizes in the nucleus with Nucleophosmin/B23 and inhibits its phosphorylation, abolishing ribosome biogenesis and rapidly resulting in apoptosis of a fraction of NSCLC cells [40]. The results presented in this paper extend the described mechanism of action and suggest that the inhibition of survival pathways may be responsible for long term anti-proliferative effects.

Refractory tumors to cisplatin therapy remain as one of the main challenges in the treatment of NSCLC patients. New second-line therapies are needed in order to improve the patient outcome. Here we generated an in vitro well-characterized cisplatin resistance model which mimics the clinical setting of patients resistant to cisplatin-based therapies [36]. The resistant cell line showed lower total levels of p65, but the pathway was highly inducible upon cisplatin exposure, in contrast with the parental A549 cell line that exhibited the opposite pattern. This result is consistent with previous reports indicating that NF-κB nuclear translocation and DNA binding is induced after the treatment with other chemotherapeutic agents in NSCLC cell lines [48]. Moreover, it has been also described that several NF-κB inhibitors lead to an increased therapeutic efficacy of cisplatin [50]. In our model, the activation of NF-κB after cisplatin treatment was a consequence of the chronic cisplatin exposure, and may be necessary for the acquisition of resistance. Although unexpected, the decrease in p65 total levels might result from a high turnover caused by chronic activation of the pathway due to the constant presence of cisplatin.

Remarkably, the cisplatin-resistant cell line resulted more sensitive to CIGB-300 than the parental one. Although CIGB-300 concentration is high, it has still clinical relevance, as can be inferred from previous in vivo assays where high doses (up to 40 mg/kg/day) administered intravenously were safe, well tolerated and non-toxic, with biodistribution values up to 1.5% of peptide uptake at tumor site 24 h post intravenous injection [64].

The sensitization to CIGB-300 may be partially due to the acquired dependence to the NF-κB signaling pathway caused by the previous cisplatin exposure. Nevertheless, additional mechanisms may be contributing to resistance. In this sense, the downregulation of the NF-κB transcriptional target c-MYC, which was distinctively diminished in A549-cispR cells after CIGB-300 treatment, may be involved in the mechanism underlying the increased sensitivity to CIGB-300. Furthermore, recent reports have associated c-MYC with the acquisition of cisplatin resistance on A549 cells, while its blockade increased apoptosis rate [65].

Therefore, CIGB-300 treatment may be considered for refractory patients after standard cisplatin-based chemotherapy in a sequential scheme of treatment. Nevertheless, more preclinical data are needed before addressing CIGB-300 as a second-line therapeutic approach after cisplatin in prospective trials.

## Conclusions

We demonstrate that CIGB-300 induced an anti-proliferative response in NSCLC models. This effect was accompanied by the inhibition of the NF-κB pathway, which was associated with an enhanced proteasome activity. Moreover, the NF-κB pathway appeared to be critically involved in the cisplatin- resistance of A549-cispR cells, which became more sensitive to CIGB-300 treatment.

In conclusion, we have successfully demonstrated that CIGB-300 modulates several CK2-dependent signaling pathways and may have a potential utility on current NSCLC therapy.

## Abbreviations

Bz: bortezomib
CI: combination index
CK2: casein kinase 2
CM: conditioned media
IC_50_: inhibitory concentration 50
MTS: 3-(4,5-dimethylthiazol-2-yl)-5-(3-carboxymethoxyphenyl)-2-(4-sulfophenyl)-2H-tetrazolium
NF-κB: nuclear factor kappa B
NSCLC: non small cell lung cancer
PI3K: phosphoinositide 3-kinase
PMA: phorbol 12-myristate 13-acetate
TBB: 4,5,6,7-tetrabromo-2-azabenzimidazole
TNFα: human tumor necrosis factor-alpha
